# Structural Biology of a Major Signaling Network that Regulates Plant Abiotic Stress: The CBL-CIPK Mediated Pathway

**DOI:** 10.3390/ijms14035734

**Published:** 2013-03-12

**Authors:** María José Sánchez-Barrena, Martín Martínez-Ripoll, Armando Albert

**Affiliations:** Department of Crystallography and Structural Biology, Instituto de Química Física “Rocasolano”, Consejo Superior de Investigaciones Científicas, Serrano 119, Madrid E-28006, Spain; E-Mails: xmjose@iqfr.csic.es (M.J.S.-B.); xmartin@iqfr.csic.es (M.M.-R.)

**Keywords:** protein kinases, calmodulin-like calcium sensor, protein structure, plant abiotic stress, ion homeostasis

## Abstract

The *Arabidopsis* SOS2 family of twenty-six protein kinases (CIPKs), their interacting activators, the SOS3 family of ten calcium-binding proteins (CBLs) and protein phosphatases type 2C (PP2C), function together in decoding calcium signals elicited by different environmental stimuli. Biochemical data suggest that stable CBL-CIPK or CIPK-PP2C complexes may be regulating the activity of various substrates controlling ion homeostasis. The available structural information provides a general regulatory mechanism in which calcium perception by CBLs and kinase activation is coupled. The structural basis of this molecular mechanism and the specificity of the network is reviewed and discussed in detail.

## 1. Introduction

The identification and characterization of the molecular species involved in the mechanisms underlying abiotic stress is crucial to understand and, ultimately, to control the plant response. Such mechanisms often include the decoding of extracellular primary stimuli (such as cold, drought, salt or others) by a membrane receptor and the transfer of such information to the intracellular compartment by an array of secondary messengers. This triggers a sequence of intracellular enzymatic reactions that orchestrates the cell response. The mechanism of such transfer of information normally relays in reversible phosphorylation, which is catalyzed by kinases and phosphatases. These proteins must be under tight regulation to ensure the integration of diverse biological stimuli and the generation of appropriate cellular responses. Due to the low specificity of the kinases and phosphatases, they have evolved to display discrete scaffold domains or even short sequence motives to regulate their function [1] and to promote the co-localization of enzymes and substrates at a particular cell place. This additionally increases the specificity of the signal.

**Figure 1:**
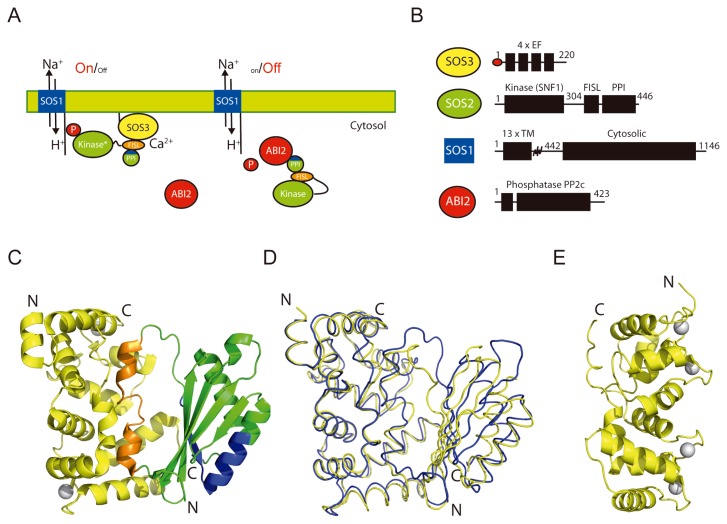
The SOS pathway is illustrative of the CBL-CIPK-PP2C network. (**A**) A schematic representation of the pathway function. (**B**) Domain structure of the protein components. (**C**) A ribbon representation of the structure of SOS3 in complex with the *C*-terminal regulatory domain of SOS2 and Ca^2+^. SOS3 is displayed in yellow, the FISL motif is displayed in orange, the PPI motif is displayed in blue, and the rest of the PPI domain of SOS2 is displayed in green. Calcium atoms are displayed as grey spheres. (**C**) The superimposition of the ribbon diagrams corresponding to the structures of the SOS3-SOS2, in blue, and CBL2-CIPK14 in yellow. (**D**) The structure of SOS3 in complex with Ca^2+^.

In *Arabidopsis*, the coordinated action of a family of ten calcium sensors (CBLs), twenty-six protein kinases (CIPKs) and phosphatases (PP2Cs), produces transient phosphorylation and the activation of several signaling pathways in response to environmental stresses [2–4]. Available data suggest a general mechanism in which stable CBL-CIPK-PP2C complexes may be regulating the activity of various ion transporters involved in stress response. Among them, the Na^+^/H^+^ antiporter SOS1 regulated by the SOS3-SOS2 (CBL4-CIPK24) complex (Figure 1A) [5,6] and the *Arabidopsis* K^+^ transporter 1 (AKT1) regulated by CBL1/9-CIPK23 complex [7]. In addition to this mechanism, it has been shown that the K^+^ transporter AKT2 activity and correct targeting to plasma membrane is dependent on its interaction with the CBL4-CIPK6 complex but independent to the phosphorylation state of the transporter [8].

The protein kinase SOS2 and the calcium sensor SOS3 are the founding members of the CBL-CIPK network [7,9–13]. SOS2 is a serine/threonine protein kinase that is required for plant salt tolerance [14–19]. SOS2 physically interacts with the calcium sensor SOS3 [20] and it is activated in a calcium dependent manner [14]. Moreover, the ability of SOS2 to interact with the protein phosphatase PP2C ABI2, suggests that SOS2-ABI2 and/or other CIPK-PP2C complexes could act as molecular on-off switches controlling the phosphorylation state of plant ion transporters. In supports to this idea, it has been shown that the 2C-type protein phosphatase AIP1 physically interacts, dephosphorylates and inactivates the AKT1 channel [7,9,10]. Despite of this knowledge there is scarce information on the effect of CBL-CIPK-PP2C interaction and/or phosphorylation on target proteins. In this sense, recent structural and functional studies including the low resolution structure of the Na^+^/H^+^ antiporter SOS1 [6,21] have shown that the docking of the SOS3-SOS2 complex induces a structural rearrangement in the SOS1 regulatory cytosolic domain. This transformation is transmitted to the transmembrane domain to activate the transporter.

Biochemical and structural studies showed that the molecular architecture of CIPKs includes the catalytic domain plus a *C*-terminal regulatory domain (Figure 1B) [17,22–24]. The structure of the CIPKs catalytic domain is unknown, but it is predicted to display the canonical Ser/Thr protein kinase fold, similar to the SNF1 kinase domains [25]. The regulatory domain can be divided functionally and structurally into a conserved 21-amino acid FISL (also known as NAF) motif and the PPI domain (Figure 1). The binding of SOS2 to SOS3 is mediated by the FISL motif, which is auto inhibitory to the kinase activity [3,17]. In addition, it is known that SOS2 can interact with the PP2C type protein phosphatase ABI2 through a *N*-terminal domain called the PPI binding motif (Figure 1) [26].

The crystal structure of the binary complex of Ca^2+^-SOS3 with the *C*-terminal regulatory moiety of SOS2 (Figure 1C) resolved central questions regarding the activation mechanism of CIPKs and the dual function of SOS2 as a kinase and a phosphatase-binding protein. The structure showed that the self-inhibitory FISL SOS2 motif bound to SOS3 is not accessible to the kinase domain. In addition, this work demonstrated that the interaction between SOS2 and SOS3 and SOS2 and ABI2 are mutually exclusive. Thus, opposite kinase and phosphatase activities cannot occur simultaneously [23,24]. On the other side of the network, the crystal structure of the calcium sensor SOS3 (Figure 1E) and the biophysical characterization of the macromolecule in solution [27,28] suggested a mechanism for the molecular function of the calcium sensor, and showed that Ca^2+^ promotes a structural reorganization of the protein. These changes are sufficient to transmit the Ca^2+^ signal to SOS2, since they involve a dramatic modification of the molecular surface properties of the macromolecule [27,28].

Despite the amount of structural information on the network at different resolution levels, there are a number of questions arising from the combined analysis of the available biochemical and structural data that deserve to be reviewed and/or remain to be investigated. Available data suggest that the mechanisms conferring signaling specificity to this network rely on: (i) the regulation of the kinase or phosphatase activities of CIPKs and PP2Cs; (ii) the differential calcium-binding affinities of the CBLs; (iii) the interaction specificity in CBL-CIPK, CBL-CIPK-PP2C complexes and CBL-CIPK-PP2C substrate combinations; (iv) the subcellular localization of all the components of the system; and (v) the differential expression pattern [29,30]. To understand most of these issues it is necessary to describe the molecular architecture of the components of the network, the interaction among them and their substrates and the effect of calcium on these interactions.

## 2. The Overall Architecture of the CBL-CIPK Complexes Provides the Structural Basis of Key Aspects of the Regulation of the Network

The crystal structures of two different CBLs in complex with the C-terminal regulatory moieties of CIPKs have been determined at atomic level [22,23]. They comprise two distinct structural domains (Figure 1). The CBL moiety and FISL motif form a single structural domain in which the calcium sensor defines a long binding cleft where the FISL motif is placed. The rest of the kinase regulatory domain, including all the residues involved in PP2C binding, constitutes the second structural domain. The overall analysis of the domain architecture of the binary complex resolves important questions about the regulation of the network. The structure shows that the CIPK autoinhibitory FISL motif and the residues involved in PP2C binding are buried in the interface between the CBL and the PPI domain. This provides a molecular mechanism for the CBL mediated activation of the CIPKs and necessarily implies that kinase activation and PP2C binding cannot occur simultaneously.

**Figure 2:**
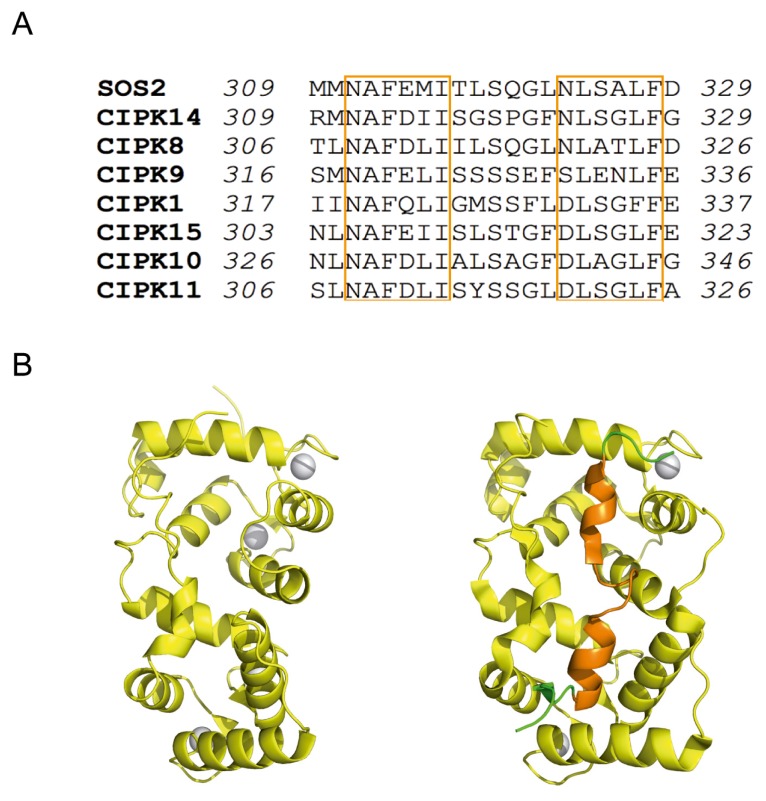
The FISL motif. (**A**) Sequence alignment of CIPK FISL motif. The α-helical regions have been highlighted with orange boxes. (**B**) Structural comparison of SOS3-Ca^2+^ in its free form (left) or bound to SOS2; the FISL motif is displayed in orange.

The topology of the CBL portion of the complex is identical to that observed for the unbound form of the known CBLs [28,31] (Figure 1). It consists of two pairs of EF hand motifs defining four potential calcium-binding sites. However, the comparison of the structures shows large differences to accommodate the FISL motif (Figure 2). The recognition mode of CBLs and the helical FISL motif is maintained by extensive hydrophobic interactions and resembles the one observed in the death-associated protein Kinase–Calcium/Calmodulin regulator complex [32] and in the regulatory and catalytic subunits of the phosphatase Calcineurin (CnB and CnA) [33]. The CBL-CIPK complex structure shows that all the calcium binding sites are solvent accessible, ensuring that CBLs can perceive calcium signals elicited by stress, bind to and activate CIPKs (Figure 1).

**Figure 3:**
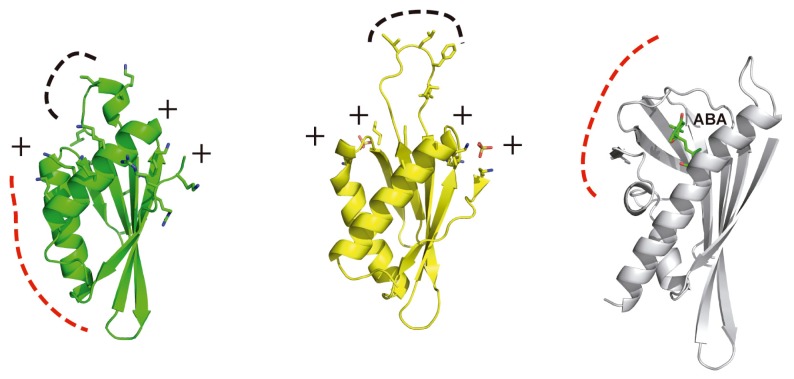
Structural comparison of SOS2 PPI domain (left) with the related structures of the KA1 lipid binding domain (middle) and PYL/PYL ABA receptors (right). Lys and Arg, as well as accessible hydrophobic residues are displayed in stick mode. The hydrophobic loop and the PP2C binding interaction area are highlighted as a black and red dashed line, respectively.

The PPI domain folds as a α/β domain in which two α-helices pack against a five-stranded antiparallel β-sheet. Interestingly, the domain topology is similar to that observed for the family of intracellular PYR/PYL ABA receptors [34,35] (Figure 3). ABA binding to these receptors leads to the activation of ABA signaling pathway by promoting the formation of stable complexes with certain PP2Cs, including the SOS2 partners ABI1 and ABI2. The structure of the PPI does not show any internal pocket to bind ABA, but this observation suggests that the PPI fold may constitute a general phosphatase binding domain; interestingly some of the residues involved in the PPI binding to PP2Cs overlap with those of the PYR/PYL ABA receptors [36,37].

Another structure function relationship is found in the striking similarity between the PPI binding domain and the kinase associated domain 1 (KA1) of septin-associated kinases (Kcc4p, Gin4p, and Hsl1p) and human MARK/PAR1 kinases [38] (Figure 3). The KA1 domains have been shown to drive these kinases to membranes by direct binding to acid phospholipids. The ability of the PPI domain to bind phospholipids has not been investigated so far, but the structural analyses may suggest this possibility. Membrane binding of the KA1 domain is mediated by positively charged patches and a hydrophobic loop that are involved in the phospholipid binding. Both the positively charged patches and the loop are present in the PPI structure. This may constitute a new mechanism for CIPKs membrane localization, which is effectively used for other kinases containing PH, C1 or C2 domains [39,40].

**Figure 4:**
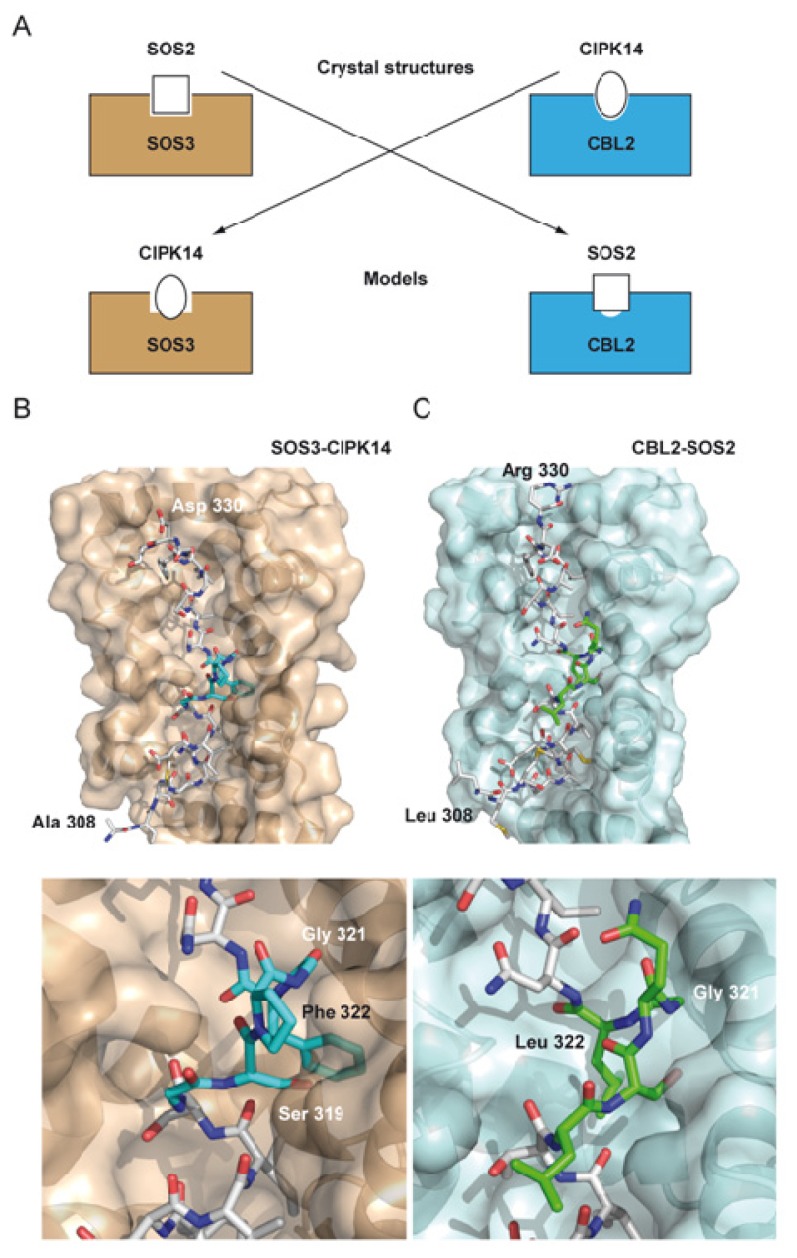
The specificity of the CBL CIPK interaction. (**A**) Schematic representation of the modeled complexes. The CIPK14 FISL moiety is inserted into the SOS3 cavity (**B**) and the SOS2 FISL is inserted into the CBL2 cavity (**C**). The lower part of the B and C panels represent a zoomed area of the FISL internal loop. SOS3 and CBL2 are displayed as a semitransparent molecular surface. The FISL motives are shown is a stick mode. Those residues form FISL overlapping CBLs are labeled.

An intriguing question is the molecular basis of the selectivity of certain CBLs towards particular CIPKs [3,17]. The joined analysis of the CIPKs amino acid sequences and the available structures provides information on this issue. The structure of the CBL-CIPK complexes shows that the FISL motif is mainly responsible of the stabilization of the complex (Figure 1). The sequence analysis among the FISL motives of CIPKs shows that only those residues involved in the loop connecting the two helical segments of the FISL are divergent (Figure 2A). Indeed, the comparison of the SOS3-SOS2 and CBL2-CIPK14 complex structures shows that the differences are maxima at this loop [22]. Interestingly, the modeled cross superposition of the SOS2 moiety onto CBL2, or CIPK14 on SOS3, shows that some of the residues belonging to the internal FISL loop overlap with the calcium sensor moiety, thus hindering the possible cross interaction between them (Figure 4). This agrees with the fact that, among the CBLs tested, SOS2 exhibited the strongest interaction with SOS3 [17].

## 3. Decoding the Calcium Signal

**Figure 5:**
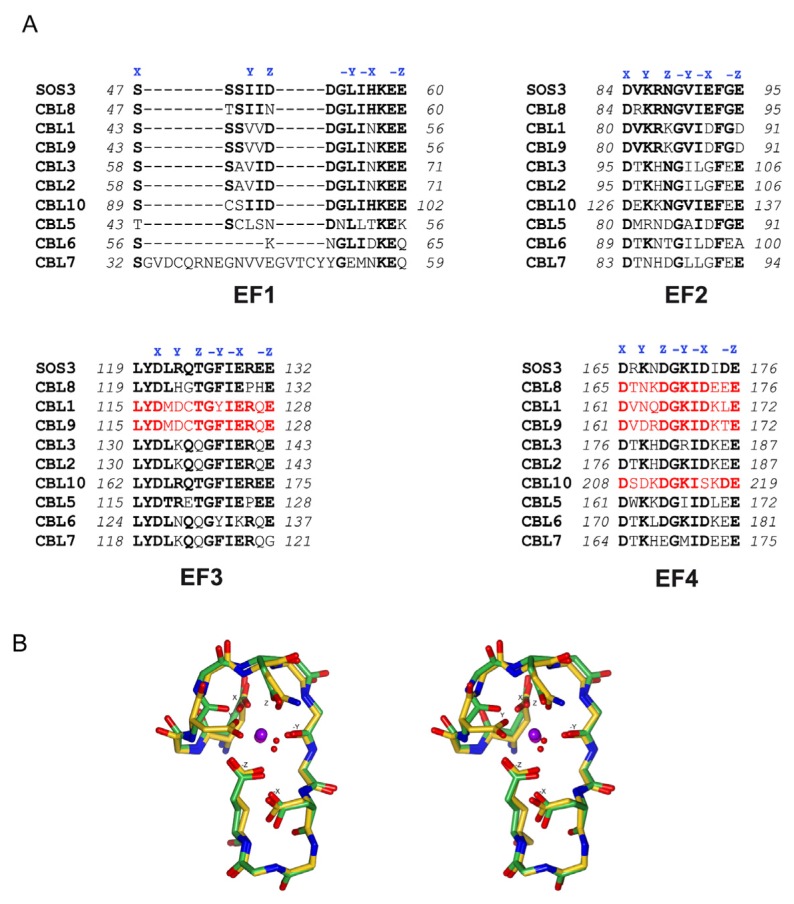
The Ca^2+^ binding EF hands of CBLs. (**A**) Sequence alignment of the AtCBL EF hands. Canonical EF hands are shown in red and residues involved in Ca^2+^ binding are highlighted by X, Y, Z, -X, -Y, -Z according to a classical EF hand. (**B**) Stereo view of the superimposition of a classical EF hand Ca^2+^ binding loop (EF-2 of CnB, PDB code 1AUI) and SOS3 EF-4 (PDB code 1V1G).

Calcium sensors contain EF hand motifs or C2 domains in order to interact with this cation. Phylogenetically, canonical EF hands constitute one of the most conserved structural elements and are responsible for high affinity Ca^2+^ binding [41]. EF hands are composed of two α-helices connected by a loop region of 12 amino acids involved in Ca^2+^ coordination. Ca^2+^ binding amino acids are located at conserved positions named X, Y, Z, -Y, -X and -Z. Amino acids at position X, Y, Z and -Z use side chain donor oxygen for Ca^2+^ interaction, a main chain carbonyl oxygen is used at position -Y, and at -X Ca^2+^ binding is mediated by a water molecule (Figure 5).

CBL proteins contain 4 EF hands like calmodulin proteins (CaMs) [42]; however, there are striking differences between them: (i) while CaMs are conserved among species, CBLs have only been identified in higher plants; (ii) CaMs can interact with a great variety of proteins, while CBLs can only interact with CIPKs; and (iii) in general, CBLs present non-canonical EF hands with a highly conserved mutation at position Y [30,41] (Figure 5).

Considering the number of canonical sites, CBL proteins can be classified in 3 groups: those containing two canonical sites (CBL1 and CBL9), those containing only one canonical site (CBL8 and CBL10) and those which do not contain any canonical site (CBL2, CBL3, SOS3, CBL5, CBL6 and CBL7) (Figure 5A) [43]. As it has been shown for other Ca^2+^ sensors such as caltractin [44], it is expected that those CBLs containing more canonical EF hand motifs display higher Ca^2+^ binding affinity. However, the crystal structures of the SOS3 and CBL2 show that Ca^2+^ saturates the four EF hand motifs despite they are not canonical. This is remarkable since EF2, EF3 and EF4 coordinating loops contain essential mutations in their sequences and EF1 loop consists of 14 amino acids instead of 12. (Figures 2 and 5) [22,23,28,31].

**Figure 6:**
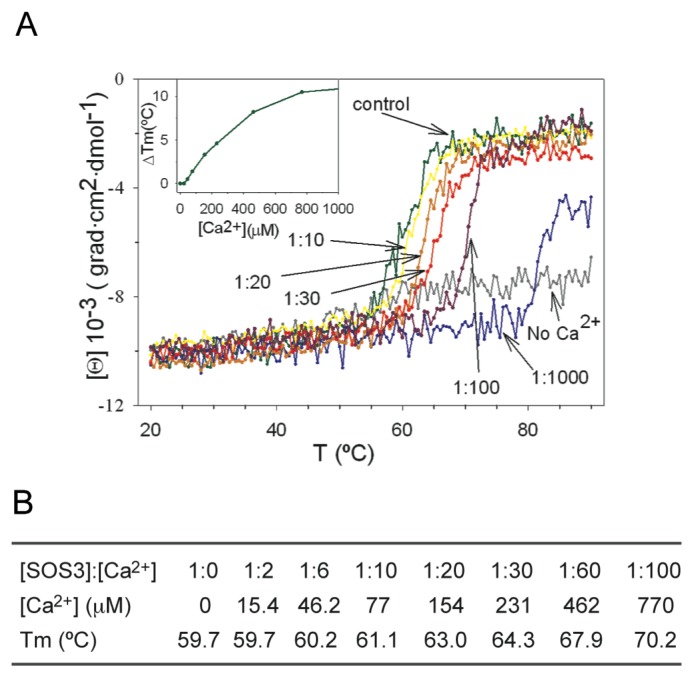
The Ca^2+^ affinity of SOS3. (**A**) Circular dichroism denaturation profiles of SOS3 as a function of Ca^2+^ concentrations. The SOS3: Ca^2+^ ratio ([SOS3]:[Ca^2+^]) is the molar ratio between protein and Ca^2+^ in the solution. Data are referred to [SOS3] = 7.7106 M. EDTA-dialyzed protein sample is labeled as “No Ca^2+^”. Inset and (**B**): Variation of the *T*_m_ (Δ *T*_m_ = *T*_m_ for a given concentration menus Tm of the control sample) with respect to the Ca^2+^ concentration.

**Figure 7:**
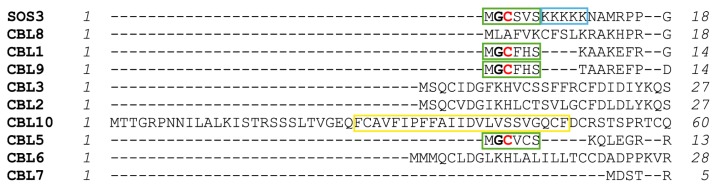
Sequence alignment of the *N*-terminal end of AtCBLs. Myristoylation motifs, polybasic sequences and transmembrane helices are outlined by green, blue and yellow boxes, respectively. Glycines/cysteines that are myristoylated/palmitoylated are highlighted in black/red bold format, respectively.

There is scant information on the Ca^2+^ affinities in CBLs. In contrast, the thermodynamics of Ca^2+^ binding to the structurally related Frequenins have been studied in detail [45]. These proteins are able to bind up to 3 Ca^2+^ ions and the calculated apparent K_d_ for such interaction is 0.7 μM. Recoverin, another closely related Ca^2+^ sensor, displays similar Ca^2+^ affinities although it only binds two Ca^2+^ ions [46]. With respect to CBL proteins, Ca^2+^ binding affinity has only been studied in SOS3 [28]. The measurement of the melting temperature (*T*_m_) of SOS3 at different Ca^2+^ concentrations estimated an upper limit of the apparent *K*_d_ for Ca^2+^ around 100 μM (Figure 6) [47]. This value is consistent with the absence of canonical EF hands in SOS3, thus it is expected that other CBLs lacking canonical EF hands would present similar Ca^2+^ affinities. These data support that the proportion of canonical/non-canonical sites among CBLs would tune distinct Ca^2+^ affinities to perceive different calcium signals. It is worth noting that the measured Ca^2+^ affinities are lower than that reported for other pant calcium sensors [48]. However, some CBLs contain *N*-terminal membrane targeting sequences that could target them close to Ca^2+^ channels where they might sense higher local Ca^2+^ concentrations (see below) (Figure 7).

Additionally, it has been shown that SOS3 displays some “non sensitive” high affinity Ca^2+^ binding sites with a structural role [47]. An extensive dialysis of SOS3 against EDTA or other chelating agents produces a general destabilization of the protein with a remarkable effect in the thermal denaturation profile (Figure 6) that can only be reversed upon Ca^2+^ addition. The rather low stability of the apoprotein suggests the existence of high affinity Ca^2+^ binding sites, from which the cation dissociates slowly. The presence of Ca^2+^ binding sites with different binding affinity has been reported for other calmodulin like proteins [45,46]. The comparative analysis of SOS3 and SOS3-SOS2 structures provides clues about the nature of the four EF sites. The second and third EF hands cannot be the structural sites since they are empty in the complex structure (Figure 1). Furthermore, fluorescence anisotropy experiments have shown that SOS3 is able to interchange ions with the media through forth EF hand, providing evidence of sensing properties [47]. Thus, we conclude that the first EF hand might be the structural binding site. Interestingly, this EF hand is highly conserved among the CBLs and displays a characteristic Ca^2+^ binding loop consisting of 14 amino acids (Figure 5).

Another interesting issue on the Ca^2+^ binding properties of CBLs arise from the fact the SOS3-SOS2 structure show that only two EF hands bind Ca^2+^ despite being able to bind up to four Ca^2+^ ions (Figures 1 and 2). The opposite is observed in CBL2 where the free form contains two Ca^2+^ ions and the complex with CIPK14 binds four. This variability suggests that only specific Ca^2+^ signals are able to promote the conformational change required to accommodate the FISL of their corresponding CIPK. A particular CBL may interact with different CIPKs depending on the number the occupied Ca^2+^ binding sites, and this in turn would depend on the calcium signature [30]. It would be necessary to undertake more thermodynamic studies in order to properly understand the Ca^2+^ sensing properties of CBL proteins.

Finally, it is also interesting to point out the ability of SOS3 to bind Mn^2+^[28]. This may be biologically relevant, since SOS2 kinase and other protein kinases from the CIPK family display enhanced activity when Mn^2+^ acts as a cofactor [2,29,49]. Manganese is an essential trace element that is required by all organisms, however, it is also potentially toxic due its redox properties. Consequently, the cell should transport sufficient quantities to a particular place without over accumulating the metal [50]. It is tempted to speculate that SOS3 or other CBL proteins could act as a carrier for this cofactor or, alternatively, could buffer the availability of free Mn^2+^ to prevent a constitutive activation of the kinase.

## 4. Structural Basis of Localization of CBL Proteins and CIPKs

In addition to the presence of EF-hands, another striking structural characteristic of CBL proteins is the presence of *N*-terminal sequences with membrane targeting motifs (Figure 7). The presence of these membrane-localization signatures is important since they would determine the localization of the CBL-CIPK complexes as it was first shown for myristoylated SOS3 [51]. SOS3, CBL1, CBL9 and CBL5 contain both palmitoylation and myristoylation sites and are located at the plasma membrane [52]. Another mechanism for membrane localization involves a single transmembrane helix at the *N*-terminal of CBL10, to target the sensor to the vacuole [12] and to the plasma membrane [11]. Additionally, SOS3 contains a region rich of lysines that could interact with phospholipids in a process called snorkeling (Figure 7) [53,54]. This polybasic sequence might be important since Ishitani *et al*. 2000 [51] showed that myristoylation is not necessary for membrane targeting but for SOS2 activation, which in turn would suggest that myristoylation might be related with the correct orientation of SOS3 with respect to the membrane.

Myristoylation might be also important in regulating Ca^2+^ binding affinities, as it has been shown for Recoverin [55] or Frequenin [45]. In both cases, the proteins display cooperativity between myristoylation and Ca^2+^ binding. The myristoyl group of Recoverin is highly sequestered in hydrophobic core of the protein in the Ca^2+^ free state and it is released upon Ca^2+^ binding. Thus, membrane binding occurs in a Ca^2+^ dependent manner [46]. It is not known if CBLs display a similar “myristoyl switch” mechanism. It might be interesting to solve the structures of the Ca^2+^ free and myristoylated CBLs and also to perform membrane binding assays of the myristoylated proteins in the presence/absence of Ca^2+^.

The CIPK localization would depend on the CBL partner. It has been proposed that SOS2 will be targeted to plasma membrane when bound to SOS3 and directed to vacuolar membrane when bound to CBL10 [11]. However, the suggested role of the PPI regulatory domain in phospholipid binding would add an additional restrain to their formation of a particular complex at cell membrane. The structure of SOS3 in complex with SOS2 regulatory domain shows that the general architecture of the complex is compatible with the dual binding of CBLs and CIPKs to the membrane since the *N*-terminal extension of SOS3 and the PPI domain of SOS2 are located at the same side of the complex (Figure 1C). This would leave the SOS3 Ca^2+^ binding sites exposed to the solvent and the SOS2 kinase domain opposite to the membrane to interact with the SOS1 antiporter [21].

## 5. Final Remarks

The mechanisms conferring specificity and regulation to the CBL-CIPK network rely on the intermolecular interactions between CBLs, CIPKs and PP2Cs and the effect of calcium ions on them. Cytosolic calcium signaling arises by a wide range of cytosolic calcium concentration elevations. These differences in absolute values may account for signaling diversification. However, calcium itself is a simple divalent cation. Consequently the signal cannot rely on a particular calcium affinity but rather, on differentially modulated affinities of many different calcium-binding proteins. The CIPKs have evolved to display discrete modules that guarantee the fine decoding on the signal triggered by extracellular stimuli. The available structural information shows that weak and transient interactions among various components of the network can decode the calcium signal and provide specificity and a sensitive regulation of cellular response to stress.

Structural data also show that CBLs are able to sense the cytosolic calcium signal elicited by stress [30,49,56,57]. However, their ability to decode particular calcium signatures is less clear. The different composition of canonic and non canonic calcium binding sites seems to control the overall calcium affinity of each sensor. However, it should be noted that the ability to buffer the cytosolic calcium concentration does not depend exclusively on this binding constant, but also on the CBL concentration [58], and this in turn, on the cellular localization and on the expression level of the macromolecule. The available structural data show that the calcium binding to CBLs promotes a dramatic conformational change in the sensor that triggers both the FISL mediated CIPK binding and the activation of the kinase, and hinders PP2C binding. Instead, *in vitro* studies on the effect of calcium in the CBL-CIPK interaction are not conclusive. While mutagenesis and low resolution structural studies shows that the formation of the CBL2-CIPK14 complex is calcium independent [22], it has been shown that the addition of calcium chelating agent to the SOS3-SOS2 complex triggers the dissociation of the complex [23].

There is a lack of structural information on the catalytic domain of the kinase and the kinase inhibition mechanism is not fully understood. Whether the unbound FISL is blocking the active site or inhibits the enzyme by an allosteric mechanism is not known. Another interesting issue is the characterization of the interaction between CIPKs and PP2Cs. Up to now there are no reported studies on the isolation of a stable complex between them. This may be a consequence of the transient and weak nature of the interaction and/or a consequence of the participation of a third molecular, yet unknown, player that could act as ABA for the interaction between PP2C to the PYR/PYL receptors [34].

The CBL/CIPK network provides a combination of calcium sensors and interacting effector kinases to provide an efficient mechanism for specific cell response to a particular calcium signal. This mechanism will not only rely on the calcium binding properties or in the kinase substrate specificity, but also in the specific combination of CBL and CIPK. The analysis of the two available structures of CBL-CIPK interacting partners showed that few amino acid modifications can control the specificity of the interaction. This would explain the promiscuity of the interaction [17] and the apparent redundancy of the function of certain CBLs to interact with a particular CIPKs [7] and prompts to the identification of additional determinants of target recognition. This would include the control of the colocalization of CBLs and CIPKs at particular cellular site. SOS3 and other CBLs are often myristoylated or palmitoylated at its *N* terminus and these modifications are important for recruiting CBLs and, consequently CIPKs, to membranes. In addition, the analysis of the regulatory domain of CIPK suggests that the PPI domain could constitute a membrane interaction module. The combination of the CBL acyl modifications and PPI domain as phospholipid-binder may function as an effective “coincidence detector” [59] promoting the binding of CIPKs to CBLs only at particular membrane locations.

The final picture providing the fundamentals the CBL-CIPK network will require a coordinated and collaborative work, integrating the characterization of different molecular species and the analysis of the system as a unique entity. This would include high and low resolution structural studies together with functional and cellular approaches.
